# Gap Opening in Graphene-Based 2D Heterostructures: The Interplay of Spin–Orbit Coupling, Hybridization, and Symmetry

**DOI:** 10.1021/acsnano.6c00354

**Published:** 2026-06-26

**Authors:** Markus Gruschwitz, Andres D. P. Unigarro, Hoyeon Jeon, Saban Hus, An-Ping Li, Sibylle Gemming, Christoph Tegenkamp

**Affiliations:** † Institut für Physik, 38869 Technische Universität Chemnitz, Reichenhainer Str. 70, 09126 Chemnitz, Germany; ‡ Center for Nanophase Materials Sciences, 6146 Oak Ridge National Laboratory, Oak Ridge, Tennessee 37831, United States

**Keywords:** epitaxial graphene, proximity coupling, spin−orbit interaction, surface transport, DFT calculations, 4-tip STM

## Abstract

Intercalating a Pb monolayer between graphene and SiC(0001) creates a densely packed metallic layer in close proximity to graphene. Using low-temperature four-point-probe scanning tunneling microscopy and density functional theory, we correlate the local conductivity of this two-dimensional heterostructure with spatially resolved spectroscopy. By varying the tunneling gap, we distinguish the density-of-states contributions of the decoupled graphene sheet and the buried Pb interface layer. At large tip–sample separations, the spectra resemble those of charge-neutral, quasi-freestanding graphene with a small contribution of the metallic Pb layer beneath. This separation confirms the presence of a 5 meV energy gap in graphene, primarily arising from symmetry breaking induced by the epitaxial Pb layer. A proximity-induced intrinsic spin–orbit coupling appears negligible or is compensated by Rashba-type interactions.

## Graphical abstract

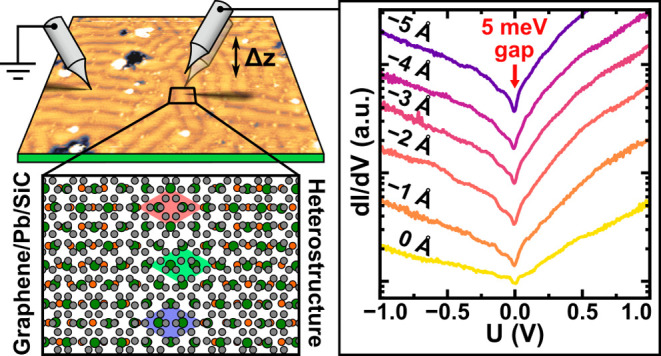

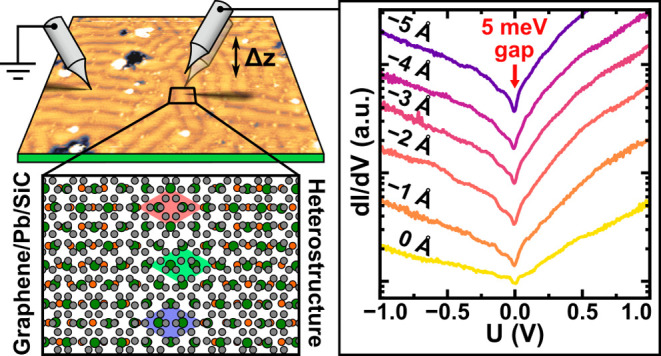

## Introduction

The stacking of two-dimensional (2D) materials enables the creation of systems with partially novel and tunable quantum properties that are becoming increasingly important in the context of quantum technologies. In this regard, proximity coupling plays a central role, critically determining the electronic properties of the resulting heterostructures.^1,2^

The extraordinary properties of two-dimensional (2D) materials were first demonstrated in graphene,^3^ which still serves as a primary starting material for 2D heterostructures and provides an excellent platform for studying proximity effects. For example, coupling with WSe_2_ induces spin–orbit coupling (SOC) in graphene,^4^ interaction with the 2D ferromagnet Cr_2_Ge_2_Te_6_ affects the quantum Hall effect,^5^ coupling with 1T-TiSe_2_ leads to charge-density waves in graphene,^6^ and interaction with CrPS_4_ can result in a quantum spin Hall effect.^7^

For an implementation of such promising concepts, it is crucial to realize atomically clean interfaces, minimize inhomogeneities, and ensure scalability. Against this background, the epitaxial approach based on graphene on SiC, followed by subsequent intercalation, appears particularly promising.^8^ With respect to intercalated systems, pioneering studies have already been carried out in the field of two-dimensional topological insulators and 2D Mott systems.^9–11^ This approach more generally opens the possibility of tuning electronic properties via the quantum geometry of these heterostructures.^12^

Moreover, the controlled opening of a band gap in graphene has attracted attention. Besides making use of quantum confinement or chemical functionalization,^13–15^ as well as the lifting of honeycomb lattice symmetry or application of strain,^16–19^ proximity coupling with 2D systems exhibiting strong spin–orbit interaction (SOI) is being intensively discussed. Specifically, the Pb-intercalated system is currently of high interest. It effectively screens substrate effects, providing epitaxially grown charge-neutral graphene.^20–22^ Moreover, a charge-to-spin conversion in graphene/Pb/ferromagnet heterostructures was recently demonstrated.^23^ The Pb-intercalated monolayer between SiC(0001) and graphene shows strong similarities to densely packed Pb monolayer structures on Si(111), exhibiting multiple structural phases, strong Rashba-split surface states, and even superconductivity.^24–27^ This makes it uniquely suited to detect electronic gap openings at the Dirac point through transport measurements.

However, the extent to which the spin-polarized surface states of the intercalated phase imprint a spin signature onto the graphene states remains unresolved. In our recent transport experiments, temperature-dependent measurements indicate that the 2D Pb monolayer phase induces a band gap on the order of approximately 5 meV.^28^ Whether this represents a true spin–orbit gap remains an open question to date. In principle, for an intrinsic spin–orbit coupling (SOC) of the Kane–Mele type, a gap of this magnitude is expected.^29,30^ However, Rashba-type SOC competes with the intrinsic effect and may even close a band gap.^31^ Furthermore, lattice symmetries and emergent moiré patterns are expected to play a crucial role. The degree of commensurability between the two 2D lattices determines the inequivalent atomic registries at the interface, which in turn can induce distinct hybridization pathways and break sublattice symmetry.^32^

In this work, we investigated this system through nanoscale low-temperature transport measurements combined with density functional theory (DFT). By precisely controlling the placement of contacts in close proximity to the tunneling tip, we are able to discriminate between contributions from the graphene layer and the buried 2D Pb layer. The directly observed band gap of approximately 5 meV is in good agreement with our previous transport results. DFT calculations further suggest the presence of spin-polarized graphene states. However, the band gap in the π-bands can be attributed to a small hybridization with the Pb states. Overall, this study demonstrates that hybridization plays a crucial role in the physics of proximity coupling in these heterostructures.

## Results and Discussion

Figure 1a shows a characteristic large-scale scanning tunneling microscopy (STM) image of a Pb monolayer intercalated between graphene and the insulating SiC(0001) substrate. We have recently discussed the origin of the observed contrast and characteristic features, such as stripes and hexagons, which arise from the coexistence of densely packed Pb(111)-like regions and strain relaxation via various types of grain boundaries.^24^ A model of the striped phase is shown in panel (b) and is intended to illustrate the epitaxial relationship between the Pb phase and the graphene layer. The DFT calculations in this work focus on the densely packed center of the stripe with Pb covering all terminating Si atoms in a Pb(1 × 1)_SiC_ reconstruction. We specifically investigate characteristic graphene arrangements on top of the dense Pb area. The so-called top (red cell), bridge (blue cell), and top-shifted (green cell) configurations of graphene w.r.t. the underlying Pb are highlighted in Figure 1b. The detailed models will be presented in Figure 4a–c. Optimized growth conditions enabled transport measurements of the 2D heterostructure on comparatively large terraces with almost no defects, revealing clear signatures of a 2D transport regime, i.e., the sheet resistance was in this case independent of probe spacing.^33^ The conductivity extracted from these measurements is 3.2 × 10^5^ S/m, in excellent agreement with previous nanoscale transport studies, as summarized in Figure 1c.^28^ Compared to our former experiments, we were able to directly measure the conductance of terraces without the need to account for barriers caused by imperfections such as large substrate steps or finite domain sizes. The extrapolation to *T* = 0 K yields a finite conductivity of approximately 3 × 10^5^ S/m, which can be attributed to the conductivity of the Pb monolayer. Assuming an electron concentration of 6.8 × 10^14^ cm^–2^ confined within an effective thickness of 0.3 nm,^21^ we obtain a carrier mobility of about 0.8 cm^2^/(V·s). This value corresponds to a scattering time of 2–3 fs and includes an effective mass of 5.3 times the free electron mass.^21^ The corresponding scattering length is in the order of 2 nm. This indicates that the grain boundary structure within the Pb monolayer acts effectively as scattering centers. Moreover, it shows that the low-temperature conductivity of the graphene layer can be neglected. In fact, as shown in the following, a small gap opens in graphene.

**Figure 1:**
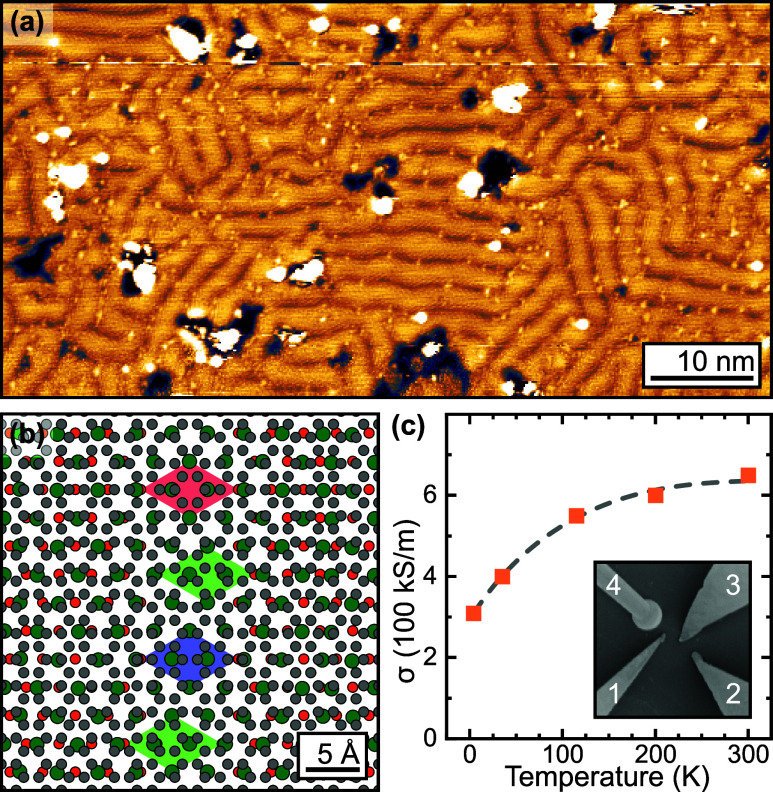
(a) Large-scale STM image (4 K, +1 V (occ. states), 50 pA) of intercalated Pb monolayer revealing the characteristic (coexisting) striped and hexagonal phases. (b) Atomistic model for the Pb atoms (green) on SiC(0001) (orange) below the graphene layer (gray). The stripes and hexagons result from the strain within the densely packed 2D Pb layer.^24^ The red, blue, and green (√3 × √3) cells highlight top, bridge, and top-shifted configurations of the Pb layer w.r.t. the graphene lattice, respectively (see also Figure 4a–c). (c) Conductivity probed at a tip spacing of *s* = 1 μm. The inset shows the scanning electron microscopy (SEM) image of the contact assembly. The conductivity value at 4 K continues the trend of previous measurements.^28^

It is unlikely that intercalation gives rise to long-range, perfectly ordered interface layers, at least on the scale of our samples. Consequently, local spectroscopic methods such as scanning tunneling spectroscopy (STS) suffer from the uncertainty of where the (Ohmic) bias contact is microscopically applied. As a result, it is *per se* difficult to make reliable layer-specific assignments of the d*I*/d*V* signals.

To overcome this limitation and, for instance, avoid charging effects and spectral shifts, we realized the Ohmic contact by employing a second STM probe positioned close to the spectroscopy probe, as shown and sketched in Figure 2a and b, respectively. The high-resolution STM image in panel (c) resolves both the graphene lattice and the characteristic features of the Pb phases, including grain boundaries. Details of the interface superstructure formed by strain gradients and resulting local Pb density variations can be found in refs 20,24,34 . On these structures, shown in Figure 2c, we acquired d*I*/d*V* maps from which site-selective averaged spectra (marked by the rectangles) were extracted, shown in panels (d) and (e) (see also Figure S1, Supporting Information). For the chosen set point, the spectrum predominantly exhibits mainly the characteristic V-shaped DOS of charge-neutral 2D graphene.

**Figure 2:**
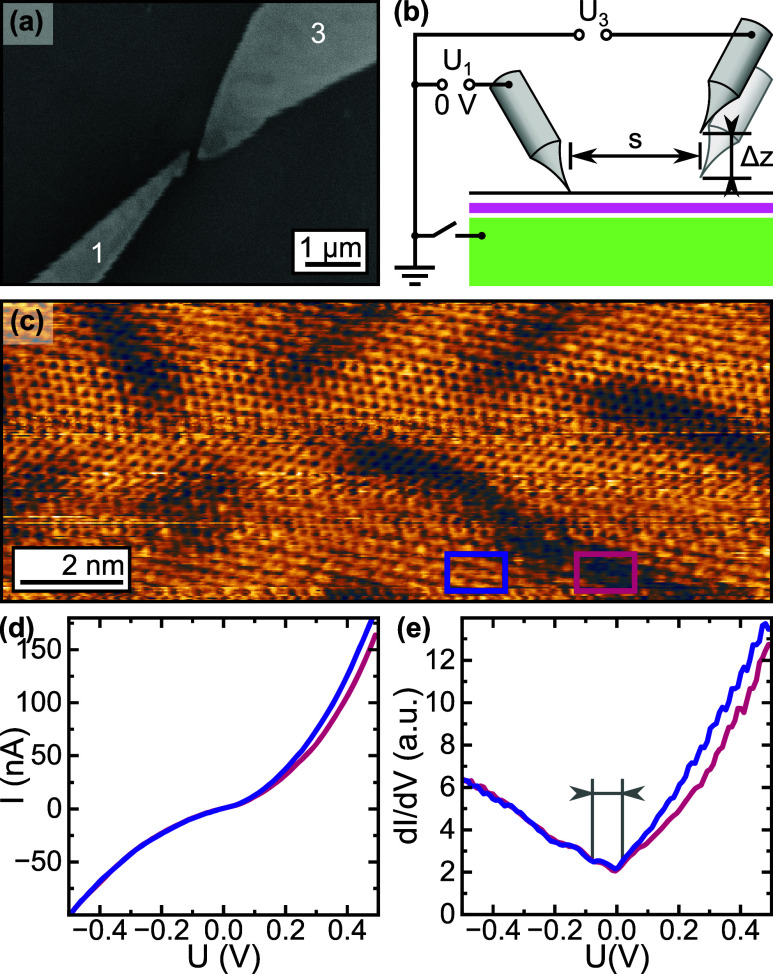
(a) SEM image showing the assembly of the grounded tip 1 and tip 3 used for STS. (b) Schematic of the tip assembly and tip selective grounding. (c) High-resolution STM image (4 K, +0.5 V, 100 pA) showing clearly the graphene lattice and Pb stripes with the grain boundaries. (d) Averaged *I*/*V* and (e) d*I*/d*V* spectra taken from d*I*/d*V* maps in the two areas marked in panel (c). The set point was +0.5 V and 100 pA, *U*_mod_ = 20 meV, thus the small gap at zero bias is not resolvable. (e) d*I*/d*V* spectra were selectively measured on top of the Pb stripes and the grain boundaries.

The suppression of the tunneling conductance near the Fermi energy, with a width of approximately 100 meV, is consistent with an inelastic tunneling gap associated with phonon excitation, as reported for freestanding graphene.^35^ Overall, the spectra show little dependence on the precise tip positions. The occupied states are largely unaffected by whether the measurement is taken on the Pb stripe or at the grain boundary. Only in the positive bias regime (unoccupied states) does a reduction in transmission appear between the two positions. Because these data were obtained from continuous d*I*/d*V* maps, the reduction directly correlates with the graphene-covered Pb monolayer structure, modifying the tunneling conditions. Even small variations in the Pb coverage and the concomitant height variation of graphene apparently affect the local work function and, consequently, the tunneling barrier height. In agreement with the metallic Pb layer in close proximity to the graphene, the d*I*/d*V* spectra show a finite offset at zero-bias voltages.^20,21,36^

To more clearly resolve the contributions from the two 2D layers and disentangle the different transport channels, we performed d*I*/d*V* spectroscopy at various tunneling gap distances, as shown in the semilogarithmic plot in Figure 3a. While the overall V-shaped spectrum characteristic of graphene (cf. Figure 2e) is present at all distances, the secondary features exhibit a pronounced dependence on Δ*z*. The relatively different positions were obtained from *I*(*z*)-curves (see panel (b)). For the reference spectrum obtained at a set point of +1 V and 1 nA (Δ*z* = 0), the spectrum is nearly perfectly V-shaped, as expected for charge-neutral graphene. Upon gradually lowering the tunneling distance, the zero-bias offset increases exponentially (Figure 3e), and additional faint peak structures at around −0.2 and −0.7 V become visible, as marked in panel (a). We attribute these peaks to electronic states of the Pb interlayer.^21,37^ As discussed further below in the context of the DFT results, the Pb states appear relatively broad and only weakly structured due to their intrinsic band structure, as well as the averaging over different configurations of the Pb lattice relative to the graphene lattice.

**Figure 3:**
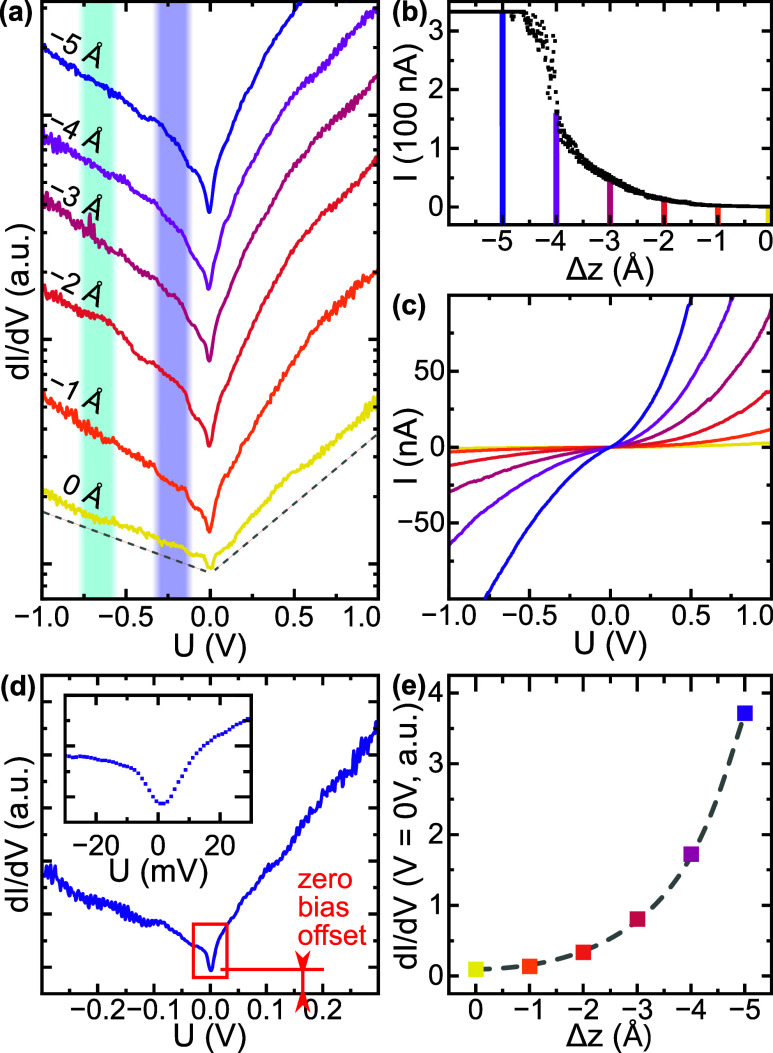
(a) STS (semilog plot) taken at the same location but for various tunneling gaps Δ*z* relative to the tunneling gap defined by the set point (+1 V, 1 nA, *U*_mod_ = 5 meV). The bars highlight the positions at which a faint peak structure appears upon reducing the tunneling distance. (b) *I*(*z*) curve to calibrate the relative tunneling gaps. (c) Corresponding *I*(*V*)-curves. (d) Zoom of a d*I*/d*V* spectrum (Δ*z* = −0.5 nm) showing the finite offset and a gap in the order of 5 meV. (e) Plot of the offset at zero bias as a function of the vertical displacement Δ*z*. The color coding is consistent across all panels.

Because the experimental geometry of our probes ensures an Ohmic contact to both 2D layers, reducing the tunneling gap increases the probability of electron injection into the Pb interface layer. Interestingly, this also implies that the graphene layer exhibits higher conductivity than the Pb layer, despite the latter having a carrier concentration larger by orders of magnitude.^24^ This finding emphasizes the exceptionally high mobility of carriers in graphene, which persists even in the presence of a slight buckling of the film.

Most striking is the small gap observed under low-bias conditions (Figure 3d). The gap magnitude is about 5 meV, in excellent agreement with values obtained from temperature-dependent transport measurements.^28^ For this configuration, where structural imperfections can be excluded by the second tip, ensuring local grounding, the gap is likely of electronic origin. The 2D heterostructure with graphene on top leads to a separation of the electronic states in real and momentum space; thus, we attribute the gap to a band gap opening in the Dirac states of graphene.

To further investigate the origin of the electronic gap, DFT calculations were performed. From our high-resolution STM measurements and structural analysis (see Figure 1a and also ref 24) we identified different arrangements of graphene relative to the Pb atoms below are present. According to the model shown in Figure 1b, three distinct translational configurations of graphene on the Pb monolayer were considered here to simulate the electronic structure of the 2D heterosystem. In the first configuration, referred to as the top configuration (see Figure 4a), two carbon atoms of graphene are positioned directly above the intercalated Pb atoms, while the remaining four occupy the three distinct bridge sites between neighboring Pb atoms. For the second configuration, shown in Figure 4b, the Pb atoms located at the corners of the unit cell occupy bridge sites with respect to the graphene lattice, and the other two Pb atoms are bridged by C–C bonds such that an almost maximally asymmetric Pb–C geometry results. A third configuration (top-shifted) considers graphene slightly shifted from the top arrangement, causing a reduction of mirror symmetries in the (√3 × √3)_SiC_ cell.

**Figure 4:**
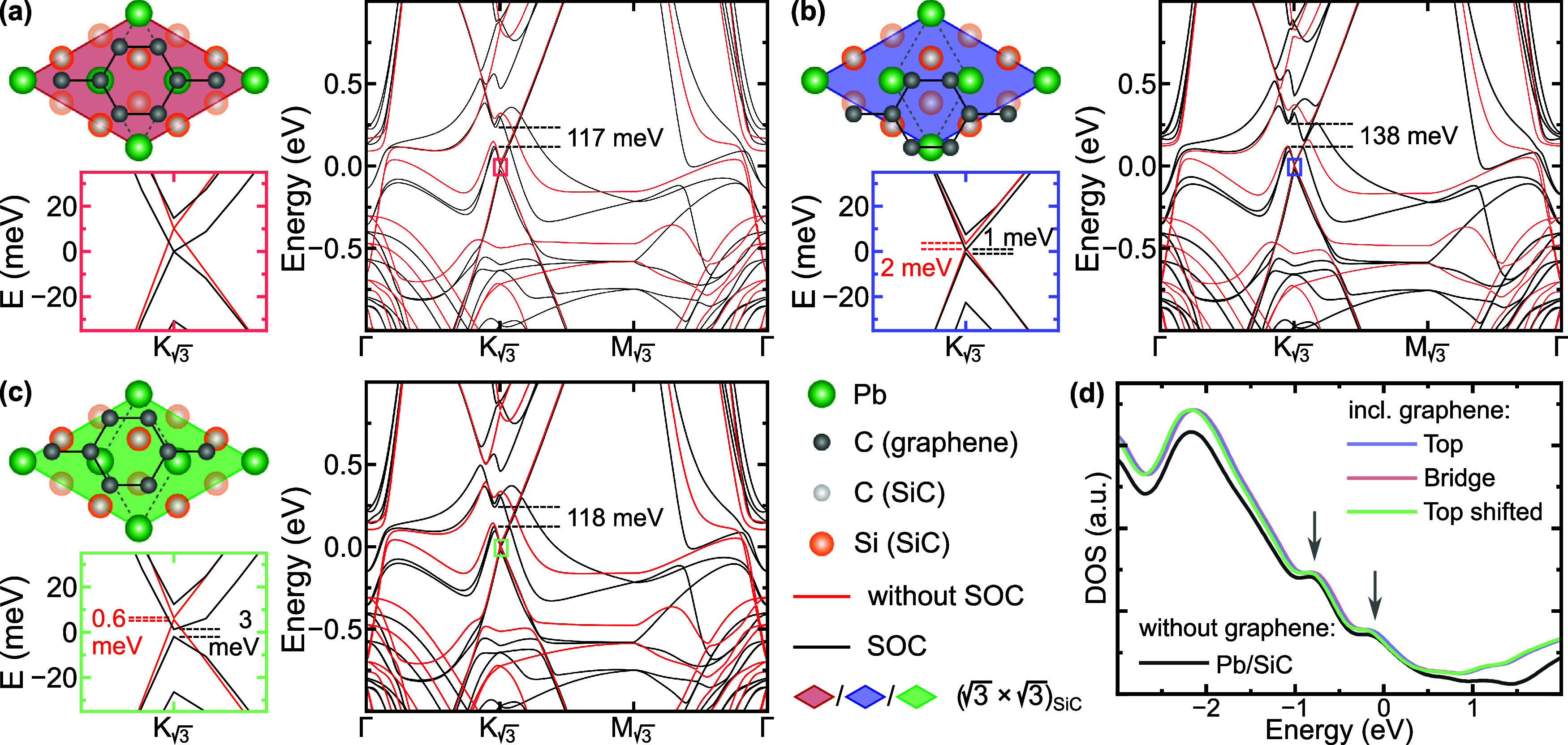
(a–c) Electronic band structures of characteristic top, bridge, and top-shifted, including ball and stick models of the graphene arrangement w.r.t. the Pb(1 × 1)_SiC_ interface. Corresponding magnifications of the regions marked by boxes around the Dirac cones of graphene are shown below the model. (d) DFT-calculated density of states (DOS) of the intercalated structure in the top (red), bridge (blue), and top-shifted (green) configurations. For comparison, the DOS of the Pb/SiC system without the graphene overlayer is also shown (grey). Due to the chosen smearing parameter, the small band gap is not visible.

The DFT results for the intercalated heterostructure in the top configuration are shown in Figure 4a. Orbital hybridization between graphene and Pb p_*z*_ orbitals (see Figure S3a, Supporting Information) leads to a noticeable distortion of the graphene linear bands around 0.17 eV above *E*_F_ along the Γ–K_√3_ direction.^38^ In this region, an avoided crossing forms, resulting in an energy gap of 117 meV. Additionally, the inversion symmetry breaking in the graphene layer, combined with the proximitized spin–orbit coupling (SOC) from the Pb layer, produces a splitting in both Dirac cones. The calculated splittings are 30 meV for the lower cone and 19 meV for the upper cone. However, no electronic gap is present at the Dirac point, which is the regime probed in our electronic transport experiments for charge-neutral graphene.

For the bridge configuration as shown in Figure 4b, the band structure exhibits several similarities to the first case. A slightly larger gap of 138 meV appears along the Γ–K_√3_ direction due to orbital hybridization (see Figure S3b, Supporting Information). Also, band splittings of around 7 and 22 meV are observed in the upper and lower Dirac cones, respectively. Interestingly, and in contrast to the aforementioned case, for the bridge configuration, a gap of 1 meV opens between the two Dirac cones.

The top-shifted arrangement in panel (c) was achieved by shifting the top arrangement. While the general band structure persists and the hybridization gap is only slightly increased (118 meV) compared to the top configuration, the Rashba-induced band splitting at the Dirac point is reduced to around 14 meV for the upper cone and 28 meV for the lower. This reduction is sufficient to open a gap of 3 meV. In contrast to the bridge arrangement, this scenario stabilizes a larger gap when including SOC.

In order to clarify the origin of the band gap of the π-bands of graphene at *E*_F_ calculated for the bridge configuration, band structure calculations without including SOC were performed as well (see Figure 4b). The results show that the gap at the former Dirac point persists with a value of 2 meV, even in the absence of SOC, indicating that it arises from the interaction with the 2D Pb layer substrate rather than from relativistic effects. A possible mechanism for the emergence of a gap at the Dirac point is sublattice symmetry breaking, which introduces a mass term in the effective low-energy Dirac Hamiltonian.^39^ However, in both the top and bridge configurations, the two carbon sublattices experience, on average, similar electrostatic potentials, i.e., no net sublattice symmetry breaking occurs. Therefore, the gap must originate from differences in the local bonding configurations between graphene and the Pb layer. In the bridge configuration, a larger fraction of C atoms are positioned closer to the Pb atoms than in the top configuration, enhancing the orbital hybridization between Pb and graphene near the Fermi level and thereby locally breaking the sublattice symmetry. As a result of this hybridization, a gap opens at the Dirac point.^40^ The top-shifted arrangement, in contrast, does break the sublattice symmetry, resulting in a further increase of the gap size. These findings highlight the sensitivity of graphene electronic structure to local stacking variations and demonstrate how changes in the relative positioning between graphene and the underlying substrate can modulate the gap formation.

The DOSs of the intercalated structures are presented in Figure 4d. To reproduce the experimental findings shown in Figure 3a, a smearing parameter of 0.1 eV was applied. As a result, the small electronic gap at zero bias is not visible in Figure 4d. The results indicate that the top, bridge, and top-shifted configurations exhibit nearly identical DOS profiles. The theoretical calculations reveal two prominent peaks in the occupied region, located at −0.7 and −0.15 eV. A direct comparison with the DOS of the Pb/SiC system without the graphene overlayer clearly indicates that these peaks originate primarily from states associated with the Pb/SiC interface and are only weakly affected by the presence of graphene. Moreover, the orbital-projected band structure (see SI) confirms that bands with significant Pb character appear around these energies. Therefore, in agreement with the STS measurements presented in Figure 3a, both peaks can be attributed to the Pb monolayer. The energy separation between the two peaks obtained from our DFT calculations (0.55 eV) is slightly larger than the experimentally observed value (0.5 eV). This discrepancy likely arises from the use of an approximate structural model of the intercalated system, which may not fully capture all structural and electronic effects present in the real material.

## Conclusions

In this study, we investigated the low-temperature transport properties of epitaxial graphene/Pb heterostructures at 4 K and correlated scanning tunneling spectroscopy (STS) measurements with density functional theory (DFT) calculations that explicitly account for spin–orbit coupling, symmetry breaking, and orbital hybridization. Charge-neutral graphene exhibits an energy gap of approximately 5 meV at 4 K, in quantitative agreement with values extracted from temperature-dependent transport measurements.^28^ Comparison with theory allows us to largely rule out a gap opening of the spin-degenerated Dirac bands driven by Pb-enhanced intrinsic (i.e., Kane–Mele type) spin–orbit coupling in graphene. It appears that a strong Rashba splitting of the Pb states induces a Rashba-type spin polarization in the π-bands of graphene. Ising-type spin components, on the other hand, seem to be absent and therefore cannot give rise to an intrinsic band gap opening in graphene.^41^ Instead, any apparent spin-related features in the π-bands originate from hybridization with Pb p_*z*_ states located above the Fermi energy. The observed gap at the Dirac point is primarily attributed to local incommensurability between the Pb and graphene lattices, which leads to local sublattice symmetry breaking and spatially varying hybridization strengths.

## Materials and Methods

Intercalation of Pb was achieved by depositing Pb multilayers at room temperature on zero-layer graphene on 6H-SiC(0001) substrates, followed by postannealing at 770 K for 5 min, as detailed in ref 24. The surface structures were previously characterized in our laboratories using high-resolution low-energy electron diffraction (SPALEED), scanning tunneling microscopy (STM), and scanning electron microscopy (SEM). Low-temperature transport and spectroscopy measurements were carried out at ORNL with a 4.6 K four-point probe STM. Local tunneling spectroscopy (STS) with high resolution was performed using a lock-in amplifier at 800 Hz with a modulation voltage of 5 meV. Nanostructured PtIr tips were employed as probes for both STM and transport experiments. For the STS measurements, the surface was grounded by a further STM tip positioned closely to the tip used for STS, i.e., occupied states are probed by applying positive voltages. Nevertheless, for the sake of a more consistent description with the literature, we also present the data here such that negative voltages correspond to the occupied states and positive voltages to the unoccupied states.

Structural relaxations and band structure calculations were conducted using density functional theory (DFT) with the ABINIT code.^42^ The exchange–correlation potential was treated within the generalized gradient approximation using the Perdew–Burke–Ernzerhof (PBE) functional,^43^ together with fully relativistic norm-conserving pseudopotentials. Geometry optimizations were performed on an 8 × 8 × 1 Monkhorst–Pack mesh until all atomic forces were below 0.0025 eV. Dispersion interactions between layers were included through the D3 van der Waals correction.^44^ For self-consistent ground-state calculations, the same 8 × 8 × 1 *k*-point grid was employed, with an energy cutoff of 1088 eV and a total-energy convergence criterion of 2.7 × 10^–8^ eV.

The intercalated structure was modeled using a commensurate approximation, where a (2 × 2) graphene supercell was matched to a (√3 × √3) SiC supercell by applying a strain of approximately 8% to the graphene layer.^45–47^ At the interface between the two materials, a Pb monolayer with (1 × 1)-SiC periodicity^37,48^ was introduced. The dangling bonds at the bottom of the SiC substrate were passivated with hydrogen atoms, and a vacuum spacing of 15 Å was included to prevent interactions between periodic images. A detailed description of the atomic structure is provided in the Supporting Information.

To facilitate a direct comparison with the experimental data, an energy shift of 0.38 eV (0.37 eV for the bridge configuration and 0.38 eV for the top-shifted configuration) was applied to the computed band structures and density of states (DOS), so that the graphene Dirac point aligns with *E* = 0. This empirical shift is based on the well-known limitations of standard density functional approximations to describe interfacial charge transfer in van der Waals systems.^49–51^

## Supplementary Material



## Notes

Details of the STM topography for graphene with intercalated Pb; details of the DFT calculations (PDF)
