# Supplementary material for: Gap Opening in Graphene-Based 2D Heterostructures: The Interplay of Spin–Orbit Coupling, Hybridization, and Symmetry
Source: ACS Nano. 2026 Jun 26;20(27):19202–8. doi: 10.1021/acsnano.6c00354 (PMC13374496; doi:10.1021/acsnano.6c00354)
Supplement: Supplementary file 1 [file nn6c00354_si_001.pdf]

Supplement Information: Gap opening in graphene-based 2D heterostructures: the interplay of spin-orbit coupling, hybridization and symmetry

Markus Gruschwitz,† Andres D.P. Unigarro,† Hoyeon Jeon,‡ Saban Hus,‡ An-Ping Li,‡ Sibylle Gemming,† and Christoph Tegenkamp*,†

†Institut für Physik, Technische Universität Chemnitz, Reichenhainer Str. 70, 09126 Chemnitz, Germany

‡Center for Nanophase Materials Sciences, Oak Ridge National Laboratory, Tennessee 37831, United States

E-mail: christoph.tegenkamp@physik.tu-chemnitz.de

STM

Fig. S1a provides a detail of Fig. 1a and emphasizes the structural variation across the striped phase as indicated by the model in Fig. 1b. The bright ridges host a considerable intensity modulation across neighboring graphene hexagons compared to the dark valleys. Along these bright stripes threefold and sixfold symmetric features appear locally. Fig. S1 (c) and (d) contain the distribution of tunneling conductance summed over the denoted voltage ranges. Only the unoccupied states reveal a reduction of tunneling conductance along the valleys between stripes as seen in the corresponding topography map in (b).

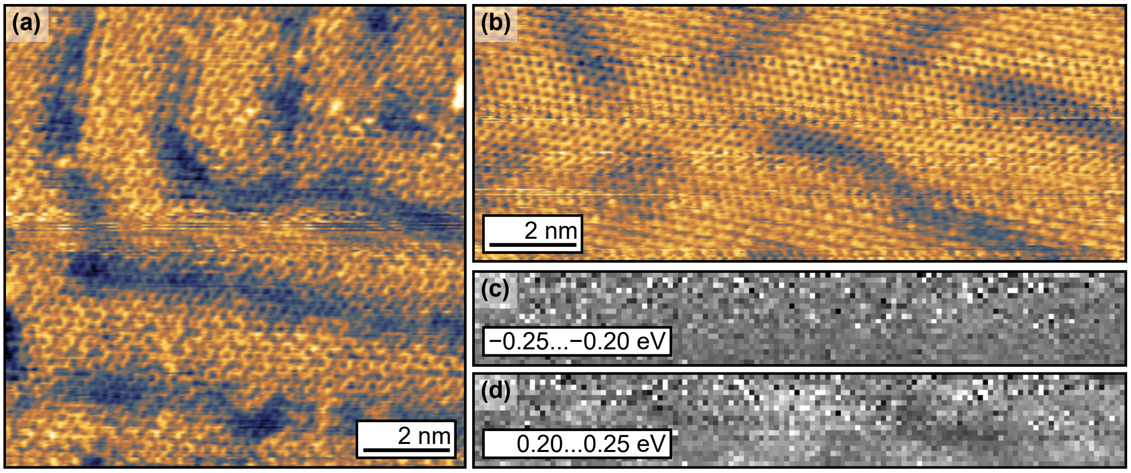

Figure S1: (a) The variation of relative positions of the Pb and graphene lattices become apparent from a variation of superstructures across the striped structure (0.5 V, 50 pA). (b) Topography at mapping position equivalent to Fig. 2c. (c) and (d) show dI/dV maps summed over the denoted voltage ranges.

DFT

Figure S2 displays the structural parameters obtained after performing DFT structural relaxations for the intercalated structure. As described in the Materials and Methods section of the main text, an approximate model consisting of a 2×2 graphene layer on a $\sqrt{3} \times \sqrt{3}$ Pb/SiC substrate was employed.

As a result of the structural optimization, a buckled Pb monolayer is obtained between the SiC substrate and the graphene layer. This buckling can be attributed to compressive strain arising from the high density of Pb atoms.[1] The interlayer distances obtained here are in close agreement with values reported in previous studies.[2–4]

Structural relaxations were performed only for the top configuration. To directly assess the effect of the relative positioning of the graphene layer with respect to the intercalated Pb atoms, the same interlayer distances obtained in the top case were used for the bridge and top-shifted configuration. The Pb atoms closest to the graphene layer are located at the corners of the unit cell shown in Fig. 4(a-c) of the main text. In the top configuration, these atoms are positioned beneath the center of the graphene hexagon, whereas in the bridge configuration they are located below a C–C bond.

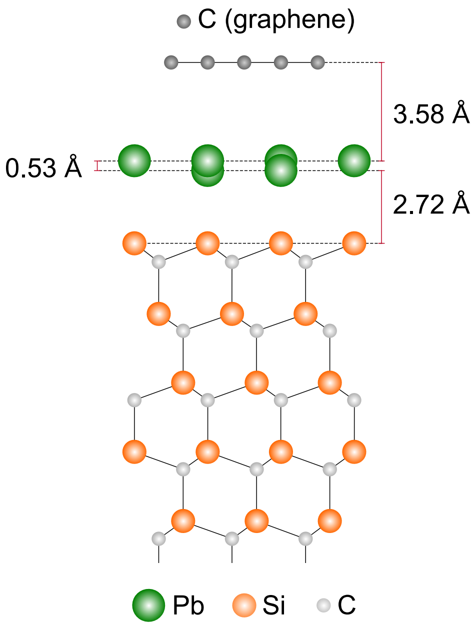

Figure S2: Ball and stick model showing the side view of the intercalated system considering the graphene layer positioned in the top configuration discussed in the main text.

To facilitate the interpretation of the band structures presented in Fig. 4(a-c) of the main text, orbital-projected band structures were calculated and are shown in Fig. S3. In these plots, the same energy shifts specified in the Materials and Methods section of the main text were applied. The top-shifted arrangement reveals the same appearance and is not shown separately. From Fig. S3, the linear dispersions associated with graphene can be clearly identified. However, a gap in the linear dispersion is observed around 0.25 eV along the Γ–$K_{\sqrt{3}}$ direction. This gap originates from orbital hybridization, as the corresponding bands exhibit significant contributions from both graphene states and the Pb p orbitals. In the bridge configuration, the Pb atoms are located closer to the C–C bonds, which enhances the hybridization effects. As a consequence, the resulting avoided crossing is approximately 20 meV larger than in the top configuration. Moreover, this enhanced hybridization leads to the emergence of a gap at the Dirac point.

At lower energies, additional orbital hybridization is observed between the SiC substrate states and the Pb p_z orbitals in both the top and bridge configurations. Around -0.25 eV, this hybridization gives rise to relatively flat bands along the Γ–$K_{\sqrt{3}}$ and $K_{\sqrt{3}}$–$M_{\sqrt{3}}$ directions.

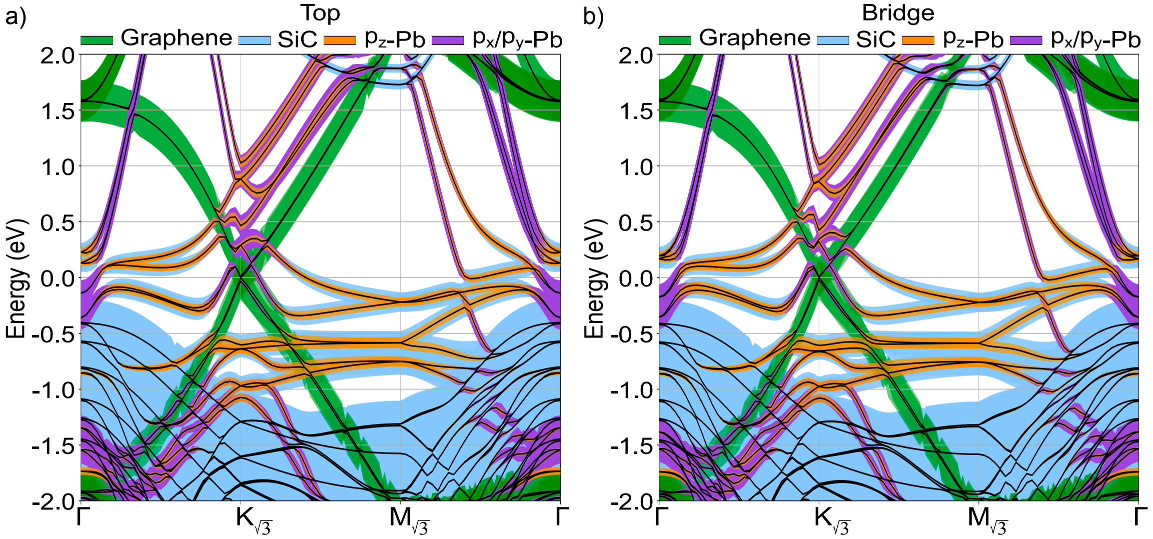

Figure S3: Orbital-projected band structure showing the contributions from graphene (green), SiC (blue), Pb p_z orbitals (orange), and Pb p_x/p_y orbitals (purple) for the (a) top and (b) bridge configurations. The same energy shifts as specified in the main text were applied.

References

1. Wang, L.-L.; Chen, S.; Kolmer, M.; Han, Y.; Tringides, M. C. Strain-modulated intercalated phases of Pb monolayer with dual periodicity in SiC(0001)-graphene interface. *Applied Surface Science* **2025**, *681*, 161572.

2. Schädlich, P.; Ghosal, C.; Stettner, M.; Matta, B.; Wolff, S.; Schölzel, F.; Richter, P.; Hutter, M.; Haags, A.; Wenzel, S.; Mamiyev, Z.; Koch, J.; Soubatch, S.; Rosenzweig, P.; Polley, C.; Tautz, F. S.; Kumpf, C.; Küster, K.; Starke, U.; Seyller, T. *et al.* Domain Boundary Formation Within an Intercalated Pb Monolayer Featuring Charge-Neutral Epitaxial Graphene. *Adv. Mater. Interfaces* **2023**, *10*, 2300471.

3. Schölzel, F.; Richter, P.; Unigarro, A. D. P.; Wolff, S.; Schwarz, H.; Schütze, A.; Rösch, N.; Gemming, S.; Seyller, T.; Schädlich, P. Large-Area Lead Monolayers under Cover: Intercalation, Doping, and Phase Transformation. *Small Struct.* **2025**, *6*, 2400338.

4. Matta, B.; Rosenzweig, P.; Küster, K.; Polley, C.; Starke, U. Pb-intercalated epitaxial

graphene on SiC: Full insight into band structure and orbital character of interlayer Pb, and charge transfer into graphene. *Phys. Rev. B* **2025**, *111*, 155435.
